# Phase- and Workload-Dependent Changes in Corticospinal Excitability to the Biceps and Triceps Brachii during Arm Cycling

**DOI:** 10.3390/brainsci6040060

**Published:** 2016-12-15

**Authors:** Alyssa-Joy Spence, Lynsey R. Alcock, Evan J. Lockyer, Duane C. Button, Kevin E. Power

**Affiliations:** 1 School of Human Kinetics and Recreation Memorial University of Newfoundland, St. John’s, NL A1B 3X9, Canada; g37ajds@mun.ca (A.J.S.); lynsey.alcock@gmail.com (L.R.A.); ejl006@mun.ca (E.J.L.); dbutton@mun.ca (D.C.B.); 2 Faculty of Medicine, Memorial University of Newfoundland, St. John’s, NL A1B 3X9, Canada

**Keywords:** motoneurone, transmastoid, transcranial, motor evoked potential, MEP, cervicomedullary evoked potentials, CMEP, cortical, muscle, central pattern generator

## Abstract

This is the first study to examine corticospinal excitability (CSE) to antagonistic muscle groups during arm cycling. Transcranial magnetic stimulation (TMS) of the motor cortex and transmastoid electrical stimulation (TMES) of the corticospinal tract were used to assess changes in supraspinal and spinal excitability, respectively. TMS induced motor evoked potentials (MEPs) and TMES induced cervicomedullary evoked potentials (CMEPs) were recorded from the biceps and triceps brachii at two positions, mid-elbow flexion and extension, while cycling at 5% and 15% of peak power output. While phase-dependent modulation of MEP and CMEP amplitudes occurred in the biceps brachii, there was no difference between flexion and extension for MEP amplitudes in the triceps brachii and CMEP amplitudes were higher during flexion than extension. Furthermore, MEP amplitudes in both biceps and triceps brachii increased with increased workload. CMEP amplitudes increased with higher workloads in the triceps brachii, but not biceps brachii, though the pattern of change in CMEPs was similar to MEPs. Differences between changes in CSE between the biceps and triceps brachii suggest that these antagonistic muscles may be under different neural control during arm cycling. Putative mechanisms are discussed.

## 1. Introduction

The basic pattern of rhythmic and alternating locomotor outputs in humans, such as arm cycling, are partially mediated via a spinally located network of neurones referred to as a central pattern generator (CPG) [1,2,3,4,5], though supraspinal input is required [6,7,8]. Studies have typically assessed spinal reflex modulation during locomotor outputs as a means to understand the neural control mechanisms underlying their production [9,10]. The results from these studies show that spinal reflexes, and thus the processing of sensory information, are both muscle- and phase- (e.g., flexion vs. extension) dependent [9,10,11]. For example, Zehr and Chua (2000) [11] demonstrated that cutaneous reflexes in some arm muscles were related to the amplitude of the ongoing background EMG (i.e., contraction intensity) while other muscles (e.g., biceps brachii) showed phase-dependent modulation.

Considerably less information is currently available regarding corticospinal excitability (CSE) modulation during locomotor outputs, though we are beginning to understand CSE modulation during leg [7,12,13] and arm cycling [8,14,15,16]. CSE can be assessed by measuring the amplitude of motor evoked potentials (MEPs) elicited via transcranial magnetic stimulation (TMS) of the motor cortex and transmastoid electrical stimulation (TMES) of corticospinal axons. Together, these measures give an indication of supraspinal and spinal excitability [8,14,16,17,18,19]. Using these techniques, Sidhu and colleagues have recently shown that CSE is phase-, muscle- and intensity-dependent to the leg muscles during cycling [7,12,13]. Work from our lab has shown phase-, task- and cadence-dependent modulation of CSE to the biceps brachii during arm cycling [8,14,15,16]. More specifically, while CSE to the biceps brachii increased throughout arm cycling as cadence increased, spinal excitability increased during elbow flexion and decreased during elbow extension [16]. Whether this occurs in different muscles during arm cycling is currently unknown.

The task-dependent neural control of arm muscles has been previously examined by assessing CSE during arm cycling compared to an intensity matched tonic contraction. These two motor outputs are compared because the generation of arm cycling is driven, in part, by spinal interneuronal networks [20]. A tonic contraction of similar muscle groups is chosen to represent a similar level of motoneurone output, but with reduced or absent activation of spinal interneuronal groups contributing to the production of arm cycling. Using this methodology, we recently showed that CSE to the biceps brachii was higher during the elbow flexion phase of arm cycling compared to an intensity-matched tonic contraction. This finding was in direct opposition to the findings of Carroll and colleagues (2006) [5], who demonstrated that CSE to the flexor carpi radialis (FCR) was higher during a matched tonic contraction compared to arm cycling. We suggested that the differences between CSE in the arm muscles may be related to the different functions of the arm muscles during arm cycling [8]. The FCR is used primarily to stabilize the wrist to allow for constant gripping of the hand pedals and is thus continuously active during arm cycling with little phase-dependence. The biceps brachii, however, demonstrates strong phase-dependence with high activation during elbow flexion to assist in propulsion and minimal activity during elbow extension occurring in the recovery phase. In addition to intermuscle differences in CSE during arm cycling, it was noted that the differences in CSE between arm cycling and tonic contraction were phase-dependent in both the biceps brachii [8] and FCR [5]. CSE, likely supraspinal in origin, to the biceps brachii was higher during the mid-elbow flexion phase of arm cycling while spinal excitability was higher at the initiation of elbow flexion [8]. Conversely, CSE and spinal excitability (assessed via the H-reflex) projecting to the FCR during arm cycling were lower at mid-elbow flexion, as was spinal excitability at the initiation of elbow flexion [5]. No studies have simultaneously assessed CSE projecting to functional antagonists during arm cycling.

The primary purpose of the present study was to determine whether CSE projecting to functional antagonists, the biceps and triceps brachii, was differentially modulated during arm cycling. A secondary objective was to assess load-dependent changes in CSE to the same muscles, also during arm cycling. We hypothesized that: (1) both supraspinal and spinal excitability to the biceps brachii and triceps brachii would be phase-dependent (i.e., higher during flexion and extension, for the biceps and triceps brachii, respectively); and (2) supraspinal and spinal excitability would increase throughout arm cycling in both muscles as load increased.

## 2. Materials and Methods

### 2.1. Ethical Approval

The procedures of the experiment were verbally explained to each volunteer prior to the start of the session. Once all questions were answered, written consent was obtained. This study was conducted in accordance with the Helsinki declaration and approved by the Interdisciplinary Committee on Ethics in Human Research at Memorial University of Newfoundland (ICEHR#: 20151928-HK). Procedures were in accordance with the Tri-Council guideline in Canada and potential risks were fully disclosed to participants.

### 2.2. Participants

Twelve male volunteers (26.3 ± 5.3 years of age, 182.7 ± 6.6 cm, 92.4 ± 17.8 kg, ten right hand dominant, two left hand dominant) partook in this study [21]. All 12 participants received TMS while 8 of those 12 received TMES (see protocols Section 2.7). Four participants did not receive TMES because the stimulation intensity required either activated nerve roots or was intolerable. Arm dominance was determined using the Edinburg handedness inventory: short form [22], to ensure that evoked potentials were recorded from the dominant arm. Given that the motor output assessed was bilateral, it was important to identify the dominant arm because of potential differences in their neural control [23,24]. Participants had no known neurological impairments. Prior to the experiment, all volunteers completed a magnetic stimulation safety-checklist in order to screen for contraindications to magnetic stimulation. Additionally, participants were required to complete a Physical Activity Readiness Questionnaire (PAR-Q+) to screen for any contraindications to exercise or physical activity.

### 2.3. Experimental Set-Up

This study was carried out on an arm cycle ergometer (SCIFIT ergometer, model PRO2 Total Body). Participants were seated upright at a comfortable distance from the hand pedals, so that during cycling, there was no reaching or variation in trunk posture. To further ensure that posture was maintained throughout all trials, each participant was strapped securely to the ergometer seat with straps placed over the shoulders and crossed over the chest. Movement of the shoulders and arms was not impeded. The hand pedals of the ergometer were fixed 180 degrees out of phase and the seat height was adjusted so that the shoulders of each individual were approximately the same height as the centre of arm crank shaft. Participants lightly gripped the ergometer handles with the forearms pronated and wore wrist braces in order to limit the movement of the wrists during cycling as heteronymous reflex connections exist between the wrist flexors and biceps brachii [25].

**Figure 1:**
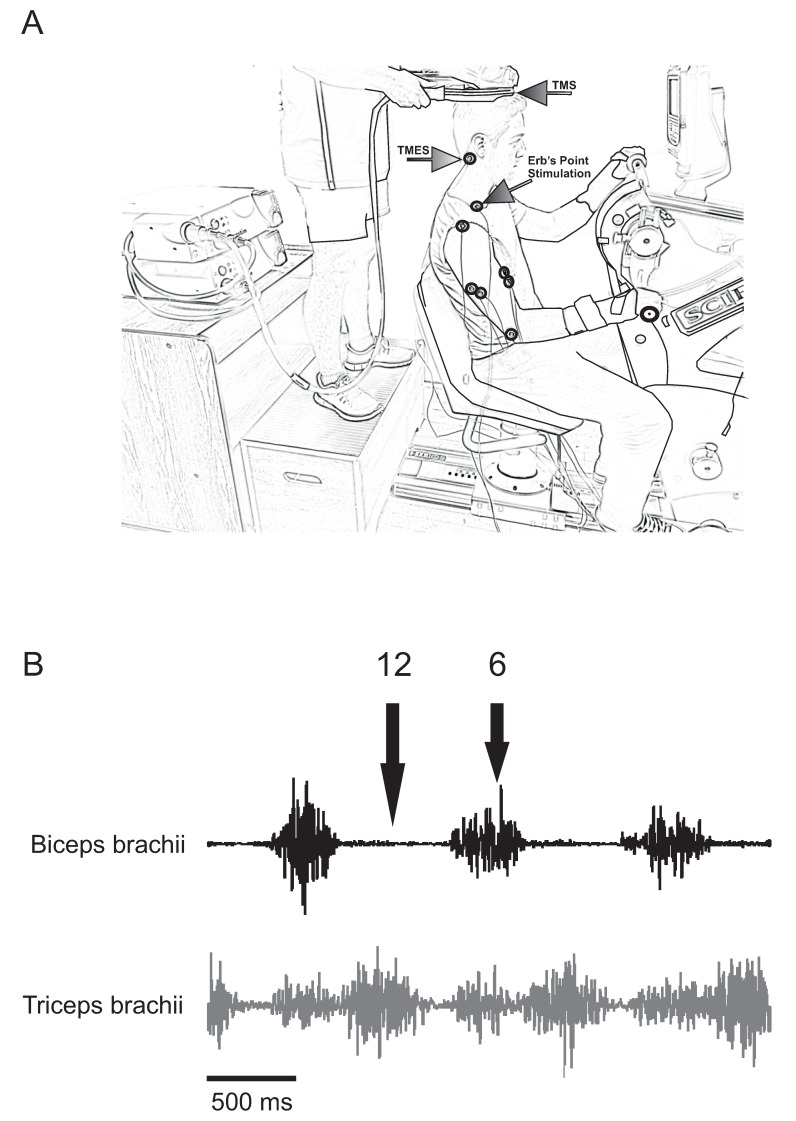
Experimental set-up. (**A**) Participants were seated with their shoulders at approximately the same height as the axis of the crank shaft on the cycle ergometer while cycling at 60 rpm at two different workloads (5% and 15% of peak power output (PPO)). Measurements were taken at the 6 o’clock (shown here) and 12 o’clock positions from the dominant arm; (**B**) Raw electromyography (EMG) trace for the biceps and triceps brachii from one participant. Note the monophasic and biphasic activation patterns of the biceps and triceps brachii, respectively. Black arrows labelled 12 and 6 represent the positions according to the face of a clock where stimulations were elicited (no stimulations in the traces shown). Abbreviations are: TMS, transcranial magnetic stimulation; TMES, transmastoid electrical stimulation.

Measurements were taken from two different locations; 6 and 12 o’clock relative to a clock face, whereby 12 o’clock was defined as the “top dead centre” of the arm crank and 6 o’clock was defined as the “bottom dead centre”. These sites were relative to the hand dominance of each individual. For example, 12 o’clock for a right handed participant would have been when their *right* hand was positioned at “top dead centre” of the arm crank (see Figure 1A; right handed participant at 6 o’clock position). For a left handed individual, 12 o’clock would have been set when their *left* hand was at “top dead centre”. These two positions were chosen as they represent periods of high (6 o’clock) and low (12 o’clock) levels of biceps brachii activation during arm cycling, with the inverse true for the triceps brachii (see Figure 1B). Movement between 3 o’clock (when the elbow reaches full extension) and 9 o’clock (when the elbow reaches maximal flexion) occurs when the elbow is flexing and the biceps and triceps brachii are most and least active, respectively. Movement between 9 o’clock and 3 o’clock occurs when the elbow is extending and the biceps and triceps brachii are less and more active, respectively. Measurements at each position were taken separately.

The study required participants to cycle at two different cycling power outputs; 5% and 15% of peak power output (PPO) as determined during a maximal sprint test (see Section 2.9). Measurements were taken at 6 and 12 o’clock for a total of four separate trials. The order of the trials was randomized and responses were triggered automatically when the arm crank passed by one of the two pre-determined positions.

### 2.4. Electromyography Recordings

EMG activity of the biceps and triceps brachii of the dominant arm were recorded using pairs of surface electrodes (Medi-Trace 130 ECG conductive adhesive electrodes) positioned over the midline of the biceps brachii and the lateral head of the triceps brachii. A ground electrode was placed on the lateral epicondyle. Prior to electrode placement the skin was thoroughly prepared by removal of dead epithelial cells (using abrasive paper) followed by sanitization with an isopropyl alcohol swab. EMG was collected on-line at 5 KHz using CED 1401 interface and Signal 5.11 (Cambridge Electronic Design (CED) Ltd., Cambridge, UK) software program. Signals were amplified (gain of 300) and filtered using a 3-pole Butterworth with cutoff frequencies of 10–1000 Hz.

### 2.5. Stimulation Conditions

Motor responses from the biceps and triceps brachii were elicited via: (1) electrical stimulation at Erb’s point; (2) transcranial magnetic stimulation (TMS); and (3) transmastoid electrical stimulation (TMES). All volunteers had prior experience with TMS, TMES and Erb’s point stimulation procedures. To determine the appropriate stimulation intensities (see Section 2.9), participants were instructed to engage in the cycling movement, but with the cycle ergometer cranks locked in place (the dominant hand pulling toward the body and non-dominate hand pushing away from the body) until the biceps brachii EMG matched a horizontal cursor set to 5% of their peak EMG recorded during the 10 s maximal sprint. Their dominant hand was placed at the 6 o’clock position and their non-dominant hand at the 12 o’clock position. The stimulation intensities for both TMS and TMES were made relative to the biceps brachii, though we also recorded from the triceps brachii as has been previously done [26].

### 2.6. Brachial Plexus Stimulation

The M_max_ of the biceps brachii was first determined by eliciting M-waves through electrical stimulation of the brachial plexus at Erb’s point (DS7AH, Digitimer Ltd., Welwyn Garden City, Hertfordshire, UK). A pulse duration of 200 µs was used and intensities ranged from 100–300 mA. The cathode was placed in the supraclavicular fossa and the anode on the acromion process. The initial stimulation intensity was set at 25 mA and gradually increased until the elicited M-waves of the biceps brachii reached a plateau. Stimulation intensity was then increased by 10% to ensure maximal M-waves (i.e., M_max_) were elicited throughout the study. Following analysis, MEP and CMEP amplitudes were normalized to the M_max_ during each trial in order to account for changes in peripheral neuromuscular propagation [19].

### 2.7. Transcranial Magnetic Stimulation

MEPs were elicited via TMS with the use of a Magstim 200 (Magstim, Dyfed, UK). Stimulations were delivered over the vertex via a circular coil (13.5 cm outside diameter). Vertex was determined by measuring the mid-point between the participant’s nasion and inion, and the mid-point between the participant’s tragi. The intersection of these two points was measured, marked and defined as vertex [8,15,18,24,27]. The coil was held tangentially to the participant’s skull, approximately parallel to the floor, with the direction of the current flow preferentially activating either the left or right motor cortex (depending on hand dominance). The coil was held firmly against the participant’s head by one of the investigators to ensure careful and consistent alignment over vertex for each trial. Stimulation intensity was started at approximately 25% of magnetic stimulator output (MSO) and gradually increased until a MEP amplitude equivalent to 15%–20% of M_max_ was found. This %MSO was used throughout the remainder of the experiment.

### 2.8. Transmastoid Electrical Stimulation

TMES was delivered using Ag-AgCl surface electrodes applied just inferior to the mastoid processes. The pulse duration was fixed at 100 µs and stimulations intensities of 125–275 mA were used (DS7AH, Digitimer Ltd., Welwyn Garden City, Hertfordshire, UK). Stimulation intensity began at 25 mA and gradually increased until the average of 8 CMEP amplitudes matched the average of the 8 MEP amplitudes previously determined [8,28]. This stimulation intensity was used throughout the remainder of the experiment.

### 2.9. Experimental Protocol

Once the intensities for Erb’s point stimulation, TMS, and TMES were determined, the four different workload trials (5% and 15% PPO at 6 and 12 o’clock) were performed. A cadence of 60 rpm was maintained for each trial, with 8 MEPs, 8 CMEPs and 2 M_max’s_ recorded at each workload and position. The order of these stimulations was randomized throughout the trial and stimulations were separated by approximately 7–8 s. To account for possible changes in the compound muscle action potential, a second trial consisting of 2 M-waves was performed immediately thereafter given that M_max_ may change over the course of an experiment [29]. These stimulations were elicited at the same workload, cadence and position as the previous MEPs and CMEPs. They were also separated by 7–8 s. These steps were then repeated for the remaining seven trials.

### 2.10. Measurements

Data was analyzed off-line using Signal 5.11 software (CED). The peak-to-peak amplitudes of MEPs, CMEPs and M_max_ of the biceps brachii were measured. The peak-to-peak amplitudes for all evoked potentials were measured from the initial deflection of the voltage trace from the baseline EMG to the return of the trace to baseline levels. Because changes in MEP and CMEP amplitudes can be the result of changes to M_max_, both MEPs and CMEPs were normalized to the M_max_ evoked during the same trial. Pre-stimulus EMG, defined as a window of the mean rectified EMG immediately prior to the stimulation artifact, was measured from the rectified traces [8]. Measurements were taken from the averaged files of all 8 CMEPs, 8 MEPs and 2 M_max_.

### 2.11. Statistics

All statistical analysis was performed using IBM’s SPSS Statistics Version 23 (IBM, Markham, Ontario, Canada. Separate two-way repeated-measures ANOVAs with factors ‘workload’ and ‘phase’ were used to assess whether statistically significant differences in MEP or CMEP amplitudes (normalized to M_max_) and the average of the pre-stimulus EMG occurred between the two cycling workloads at each phase of the cycle (i.e., elbow flexion and extension). All data were normally distributed as determined using the Kolmogorov-Smirnov test for normality. Assumptions of sphericity were tested using the Mauchley test, and if it was violated, the appropriate correction was applied (i.e., Greenhouse Geisser or Huynh-Feldt). A Bonferroni *post hoc* test was performed to test for significant differences between interactions. All statistics were run on group data and a significance level of *p* < 0.05 was used. All data are reported in text as means ± SD and illustrated in figures as mean ± SE.

In order to make inferences as to changes in supraspinal and spinal excitability during cycling it is important that the intensity of the motor output, as estimated via pre-stimulus EMG levels, in the biceps brachii and triceps brachii be similar when MEPs and CMEPs were evoked. Thus, we compared pre-stimulus EMG levels between MEPs and CMEPs, within phase (flexion vs. extension) and workload (5% or 15% PPO) using paired *t*-tests.

## 3. Results

### 3.1. Biceps Brachii

#### 3.1.1. Corticospinal Excitability to the Biceps Brachii during Arm Cycling

**Figure 2:**
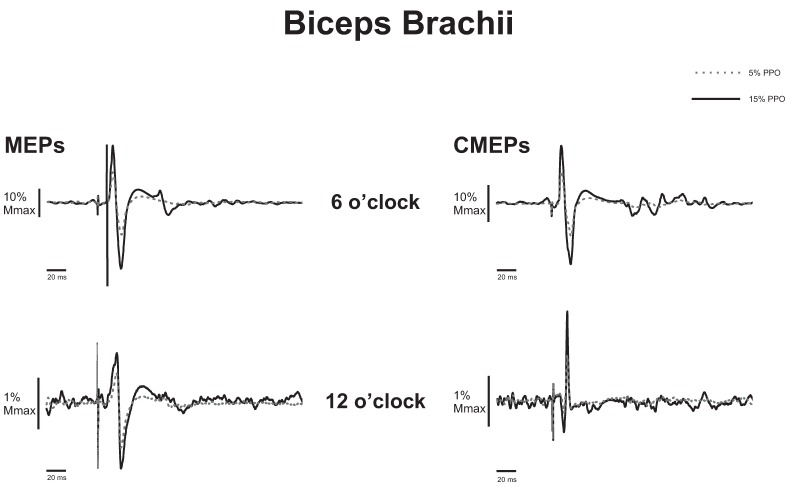
Biceps brachii representative example (*n* = 1). Average motor evoked potentials (MEPs; **left** panel) and cervicomedullary evoked potential (CMEPs; **right** panel) traces following eight stimulations during arm cycling at 5% PPO (dashed gray line), and 15% PPO (solid black line) at the 6 o’clock (**top** panels) and 12 o’clock (**bottom** panels) positions. Amplitudes are expressed as a percentage of maximal M-wave (M_max_).

**Figure 3:**
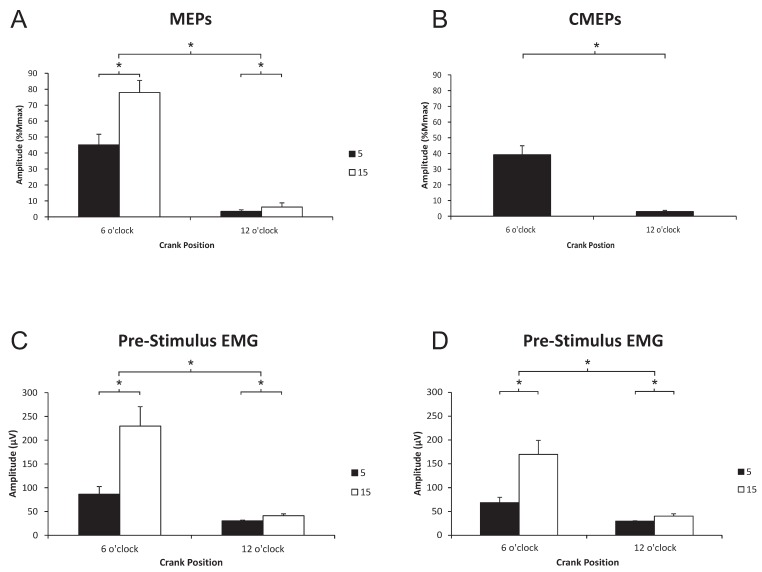
Group data (means ± SE, *n* = 12) for biceps brachii MEP amplitudes (**A**), and pre-stimulus EMG prior to transcranial magnetic stimulation (TMS; **C**). Group data (means ± SE, *n* = 8) for CMEP amplitudes (**B**) and pre-stimulus of the biceps brachii prior to TMES (**D**). MEP and CMEP amplitudes are expressed relative to the M_max_ taken during cycling at the same cadence and workload. * Significant difference (*p* < 0.05). Abbreviations are: MEP, motor evoked potential; EMG, electromyography; TMES, transmastoid electrical stimulation; CMEP, cervicomedullary motor evoked potential.

*MEP amplitude.* Figure 2 (left panel) shows the average of eight MEPs expressed as a percentage of M_max_ at 5% and 15% of PPO at the 6 o’clock and 12 o’clock positions (data is from one participant). In this example, MEPs expressed as a percentage of M_max_ at the 6 o’clock position are 22.03% and 53.2% during the 5% and 15% PPO trials, respectively. At the 12 o’clock position MEPs are 1.44% and 2.19% M_max_ during the 5% and 15% PPO trials. There were significant main effects for position (flexion > extension, *p* < 0.001), load (15% > 5%, *p* = 0.001), as well as interaction effects (*p* = 0.006). As a group, MEP amplitudes during flexion were 45.10% and 77.89% M_max_ at 5% and 15% PPO, respectively; during extension, average MEP amplitudes were 3.46% and 6.20% M_max_ at 5% and 15% PPO, respectively (Figure 3A).

*Pre-stimulus EMG for MEPs.* Significant main effects for position (flexion > extension, *p* = 0.001), load (15% > 5%, *p* < 0.001), as well as interaction effects (*p* = 0.001) were observed. As a group, pre-stimulus EMG during flexion was 86.4 and 230 µV at 5% and 15% PPO, respectively; during extension, pre-stimulus EMG was 30.5 and 41.1 µV at 5% and 15% PPO, respectively (Figure 3C).

#### 3.1.2. Spinal Excitability to the Biceps Brachii during Arm Cycling

CMEP amplitude. Figure 2 (right panel) shows an example of the differences in CMEP amplitude between 5% and 15% PPO cycling loads at 6 o’clock and 12 o’clock positions. In this example, CMEPs expressed as a percentage of M_max_ were 18%, and 1.16% during the 5% PPO trial and 31.5% and 2.42% during the 15% PPO trial. There was a significant main effect for position (flexion > extension, *p* < 0.001) with no effect of load (*p* = 0.179). As a group, CMEP amplitudes during flexion and extension were 39.2% and 3.0% M_max_, respectively (Figure 3B).

Pre-stimulus EMG for MEPs. Significant main effects for position (flexion > extension, *p* = 0.004), load (15% > 5%, *p* = 0.001) as well as interaction effects (*p* = 0.017) were observed. As a group, pre-stimulus EMG during flexion was 68.5 and 169.9 µV at 5% and 15% PPO, respectively; during extension, pre-stimulus EMG was 29.8 and 40.2 µV at 5% and 15% PPO, respectively (Figure 3D).

Background EMG of biceps brachii between stimulation types as function of workload arm cycling. Paired *t*-tests reveal no significant effect of stimulation type at any intensity or position (*p* = 0.284–0.893). Thus general comparisons between changes in MEP and CMEP amplitudes are warranted.

### 3.2. Triceps Brachii

#### 3.2.1. Corticospinal Excitability to the Triceps Brachii during Arm Cycling

**Figure 4:**
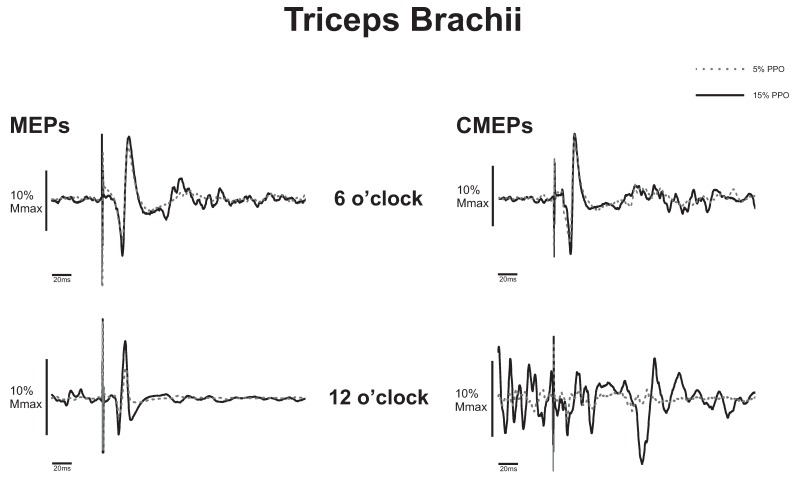
Triceps brachii representative example (*n* = 1). Average motor evoked potentials (MEPs; left panel) and cervicomedullary evoked potentials (CMEPs; right panel) traces following eight stimulations during arm cycling at 5% PPO (dashed gray line), and 15% PPO (solid black line) at the 6 o’clock (top panel) and 12 o’clock (bottom panel) positions. Amplitudes are expressed as a percentage of maximal M-wave (M_max_). Abbreviation is: PPO, peak power output.

**Figure 5:**
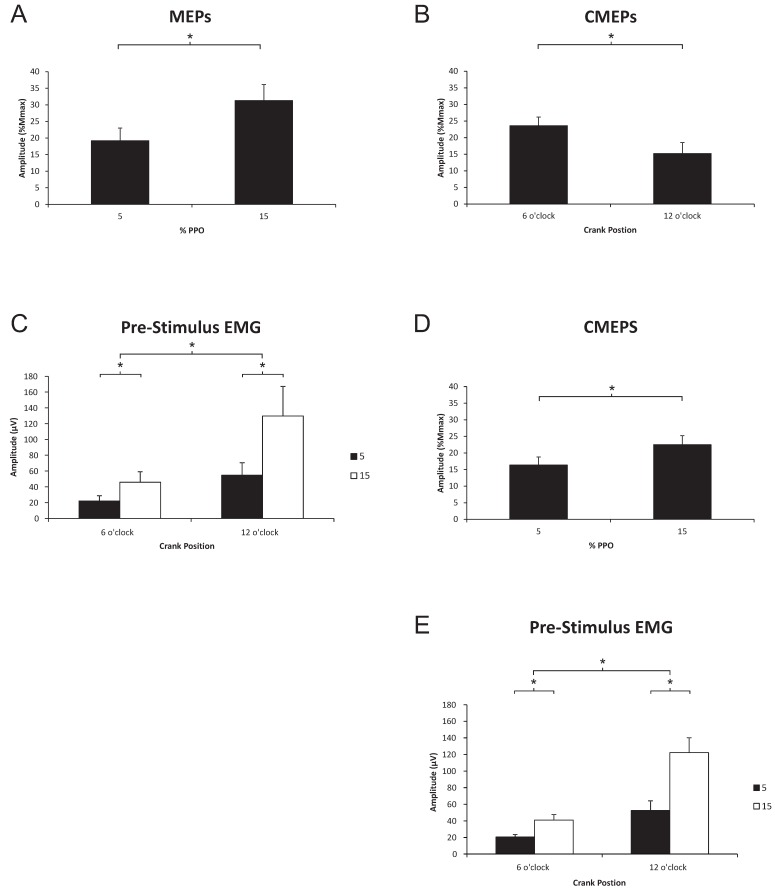
Group data (means ± SE, *n* = 12) for triceps brachii MEP amplitudes (**A**), and pre-stimulus EMG prior to transcranial magnetic stimulation (TMS; **C**). Group data (means ± SE, *n* = 8) for CMEP amplitudes based on position (**B**) and % PPO (**D**), as well as pre-stimulus of the triceps brachii prior to TMES (**E**). MEP and CMEP amplitudes are expressed relative to the M_max_ taken during cycling at the same cadence and workload. * Significant difference (*p* < 0.05). Abbreviations are: MEP, motor evoked potential; EMG, electromyography; PPO, peak power output; TMES, transmastoid electrical stimulation; CMEP, cervicomedullary motor evoked potential.

*MEP Amplitude*. Figure 4 (left panel) shows an example of MEPs elicited at 5% and 15% PPO cycling loads at the 6 o’clock and 12 o’clock positions. In this example, MEPs expressed as a percentage of M_max_ were 17.8% and 40.7% during the 5% PPO trial and 22.6% and 76.9% during the 15% PPO trial. There was a significant main effect for load (15% > 5%, *p* = 0.006), but no significant effect of position (*p* = 0.246), or interaction effects (*p* = 0.053). As a group, MEP amplitudes during were 19.2% and 31.3% M_max_ at 5% and 15% PPO, respectively (Figure 5A).

*Pre-stimulus EMG for MEPs.* Significant main effects for position (extension > flexion, *p* < 0.001), load (15% > 5%, *p* < 0.001) as well as interaction effects (*p* < 0.001) were observed. As a group, pre-stimulus EMG during extension was 54.7 and 129.7 µV at 5% and 15% PPO, respectively; during flexion, pre-stimulus EMG was 22.2 and 45.9 µV at 5% and 15% PPO, respectively (Figure 5C).

#### 3.2.2. Spinal Excitability to the Triceps Brachii during Arm Cycling

*CMEP amplitude*. Figure 4 (right panel) shows an example of the differences in CMEP amplitude between 5% and 15% PPO cycling loads at 6 o’clock and 12 o’clock positions. In this example, CMEPs expressed as a percentage of Mmax were 18.2%, and 3.81% during the 5% PPO trial and 19.8% and 8.3% during the 15% PPO trial. There were significant main effects for position (flexion > extension, *p* = 0.042) and load (15% > 5%, *p* = 0.003), and no interaction effect (*p* = 0.353). As a group, CMEP amplitudes during flexion and extension were 15.2% and 23.6% M_max_, respectively (Figure 5B). Average CMEP amplitudes were 16.4% and 22.5% M_max_ at 5% and 15% PPO, respectively (Figure 5D).

*Pre-stimulus EMG for CMEPs.* Significant main effects for position (extension > flexion, *p* = 0.002), load (15% > 5%, *p* = 0.001) as well as interaction effects (*p* = 0.005) were observed. As a group, pre-stimulus EMG during extension was 52.5 and 122.3 µV at 5% and 15% PPO, respectively; during flexion, pre-stimulus EMG was 20.6 and 41.0 µV at 5% and 15% PPO, respectively (Figure 5E).

*Background EMG of triceps brachii between stimulation types as function of workload during arm cycling.* Paired *t*-tests reveal no significant effect of stimulation type at any intensity or position (*p* = 0.69–0.988). Thus general comparisons between changes in MEP and CMEP amplitudes are warranted.

## 4. Discussion

This is the first study to report on corticospinal excitability of antagonistic muscle groups during arm cycling. As expected, corticospinal and spinal excitability projecting to the biceps brachii was higher during elbow flexion than extension and was increased with a higher relative workload. The triceps brachii, however, provided some unexpected results. First, there were no phase-dependent differences in CSE projecting to the lateral head of the triceps brachii, though CSE did increase with an increased intensity. Second, spinal excitability was *higher during elbow flexion than extension*. Thus, there are intermuscle differences in the phase- and workload-dependent changes to corticospinal excitability during arm cycling.

### 4.1. Phase-Dependent Modulation of Corticospinal and Spinal Excitability

Corticospinal and spinal excitability to the biceps brachii was significantly greater during elbow flexion than extension, a finding we have demonstrated previously [8]. The phase-dependent differences in CSE can be partially accounted for by changes in supraspinal and spinal excitability (given the same pattern of change as those in CSE, Figure 3A,B), though the exact mechanisms are not yet known. Our previous work showed that supraspinal excitability was different between cycling and tonic contraction during elbow flexion and we suggested that increased supraspinal excitability during this phase of arm cycling was to enhance the descending drive to the spinal cord to increase the recruitment and firing rates of the spinal motoneurones, thus producing adequate torque generating capabilities [8]. However, the cortical mechanisms associated with this increase in excitability have yet to be determined. At the spinal level, changes in synaptic input and/or intrinsic motoneurone properties that would act to increase spinal motoneurone excitability could also explain the larger CMEP amplitude during elbow flexion compared to extension when the motor pool is less active and likely receiving reciprocal inhibitory input from the triceps brachii motor pool [8,30].

We hypothesized that overall CSE and spinal excitability projecting to the triceps brachii would be greater during elbow extension than flexion and were thus surprised that there was no phase-dependent difference in CSE to the triceps brachii, despite the significant phase-dependent difference in the pre-stimulus EMG amplitude (i.e., EMG higher during elbow extension; see Figure 5C). This apparent dissociation between CSE and EMG suggests that changes in overall CSE assessed via TMS-evoked MEPs relate to differences in central motor command as opposed to changes in central drive required to increase EMG levels. That is, changes in MEP amplitude do not necessary relate to changes in ongoing muscle activity. This may be the case in the present study (i.e., dissociation between EMG and changes in CSE), especially given that arm cycling likely involves the operation of a spinal CPG [31] and is under different neural control than tonic contractions [8,11,14,20,32]. This also suggests that the central command controlling the triceps and biceps brachii may be different, given the phase-dependent modulation of CSE in the biceps brachii. Intermuscle differences in CSE during locomotor outputs in the legs have been previously reported [7]. Sidhu and colleagues (2012) [7] demonstrated differences in the CSE to the rectus femoris and biceps femoris compared to the vastus lateralis during leg cycling and suggested that intermuscle differences in the phase-dependent modulation of CSE was a function of biarticular versus monoarticular muscles. It is noted that arm cycling is a bilateral motor output and we did not assess the activity of the non-dominant limb. It is possible that the participants relied on elbow flexion of the non-dominant limb to produce elbow extension in the dominant limb, resulting in a lack of phase-dependency in CSE to the triceps brachii. Though we cannot rule out this possibility we consider it unlikely given that the EMG of the triceps brachii was higher during elbow extension than flexion in the dominant limb.

Even more surprising was that spinal excitability to the triceps brachii was higher during elbow flexion than extension, despite the higher pre-stimulus EMG during elbow extension (Figure 5B,E). There are several factors to consider for explaining this finding. First, higher spinal excitability during flexion than extension combined with a lack of phase-dependent modulation of CSE suggests that supraspinal excitability may be reduced to the triceps brachii during elbow flexion phase. Second, it is noted that we recorded the activity of the lateral head of the triceps brachii, a monoarticular muscle, which although active in elbow extension does not necessarily represent the activity or excitability in the other three elbow extensors (i.e., long and medial head of triceps brachii and the anconeus). The motoneurones projecting to the lateral head have lower recruitment thresholds than the long head when shoulder and elbow joint angles are 0 and 90 degrees of flexion respectively, during isometric contractions [33]. Those joint angles are equivalent to the elbow flexion position in the present study. Thus, the larger CMEPs during elbow flexion could be muscle specific and due to increased recruitment of spinal motoneurones. It is presently unclear how corticospinal and/or spinal excitability to the other elbow extensors is modulated during arm cycling.

Third, during elbow flexion the triceps brachii are in a stretched position compared to elbow extension, which would presumably increase muscle spindle activity. Increased input from Ia afferents is known to exert a strong excitatory influence on motoneurone excitability, which may lead to increased recruitment and/or firing rate by activating persistent inward currents (PICs), for example, which amplify synaptic inputs [34,35]. Wilson and colleagues (2015) [36] recently demonstrated, via indirect measures, that the contribution of PICs to motoneurone excitability was higher in the lateral head of the triceps brachii than the biceps brachii during isometric contractions. It is also noted that: (1) motoneurones with lower recruitment thresholds, such as those in the lateral head of the triceps brachii, also have a higher incidence of PICs; and (2) there is a higher incidence of PICs in extensor compared to flexor motoneurones [37,38]. It is possible that the stretch activated facilitation of PICs to the triceps brachii during elbow flexion may have increased spinal motoneurone excitability, thus increasing CMEP amplitude. The contribution of PICs to motoneurone excitability may be reduced during elbow extension when the triceps brachii are no longer in a stretched position, thus reducing PIC related amplification of synaptic input [39].

Finally, though corticomotoneuronal excitation occurs monosynaptically for both the biceps and triceps brachii, the incidence of those connections are much less in the triceps brachii, which involves a larger portion of polysynaptic connections in the corticomotoneuronal pathway [40,41]. Thus, although TMES-evoked CMEPs are suggested to represent spinal motoneurone excitability [17], CMEPs represent the ability of motoneurones to respond to synaptic input, not changes in the intrinsic properties of spinal motoneurones that are modifiable during locomotor outputs, such as the voltage threshold for action potential initiation and afterhyperpolarization amplitude [42,43,44]. With more interneurones relaying the information to the triceps brachii, TMES-evoked CMEPs in the triceps brachii are thus more heavily influenced by interneuronal excitability than the biceps brachii. Given that arm cycling has been shown to be generated, in part, via a spinally located CPG [20,45], it is likely that many last order interneurones (excitatory and inhibitory) are active [46], thus influencing motoneurone excitability as seen in the CMEP amplitudes. The relative contribution of the corticomotoneuronal pathway to various muscles during locomotor output may thus be different, with some populations of motoneurone pools receiving greater cortical input than others. It may be that the observed intermuscle differences presented in corticospinal control herein represent different, muscle-dependent neural control strategies.

One possibility that we consider unlikely to account for similar spinal excitability of the triceps brachii during elbow extension and flexion, but cannot rule out with certainty, is that the higher pre-stimulus EMG during elbow extension could have blunted the CMEP amplitude due to the fact that the motoneurone pool was already highly active (i.e., the stimulation was insufficient to activate additional motoneurones or to increase their firing rate). However, when pre-stimulus EMG is carefully considered, the pre-stimulus EMG levels during elbow flexion and 15% PPO are not significantly different from those during elbow extension and 5% PPO, yet the CMEP amplitude during flexion are much larger than those during extension and 5% PPO (see Figure 5B,E).

### 4.2. Load-Dependent Modulation of Corticospinal and Spinal Excitability

Load-dependent increases in CSE were expected and did occur in both the biceps and triceps brachii during both flexion and extension phases of arm cycling. The loads used in the present study were significantly different from each other in terms of motoneurone output as seen in the pre-stimulus EMG (see Figure 3C,D and Figure 5C,E), which is a general measure of muscle contraction intensity (i.e., the higher the pre-stimulus EMG the more active the muscle). Previous work examining the CSE to the biceps brachii during isometric contractions have reported increases in both MEP and CMEP amplitudes as the contraction intensity increases, up to a limit of approximately 60% of maximal voluntary contraction force output [18,24]. This suggests that spinal excitability contributed to the overall increase in CSE seen during these experiments. In the present experiment, significantly larger MEPs were recorded from both the biceps (Figure 3A) and triceps brachii (Figure 5A) muscles during arm cycling at 15% as opposed to 5% of PPO. Significantly larger CMEPs were recorded for the triceps but not biceps brachii at 15% vs. 5% PPO, though the changes in CMEPs in the biceps brachii followed a similar pattern changes in MEP, suggesting that spinal excitability contributed to the increase in MEP amplitude. Perhaps the most novel and interesting point to consider is that it appears as though the type of intensity may be important in determining CSE during arm cycling. As opposed to isometric contractions, one can alter the intensity of arm cycling by changing the load, cadence, or a combination of both. In the present study we show that by increasing the load, the CSE to the biceps brachii increases during flexion and extension. In our previous work, however, we used cadence to alter the intensity of cycling and demonstrated that although overall CSE was increased to the biceps brachii during both phases as cadence increased, spinal excitability actually *decreased*, suggesting an overall increase in supraspinal excitability (see Figure 4A,D, Forman et al. 2015 [16]). Triceps brachii data, unfortunately, was not assessed and there is currently no information available regarding CSE to the triceps brachii during different intensity tonic contractions.

## 5. Conclusions

The most novel finding in the present study was that the phase-dependent modulation of corticospinal and spinal excitability appears to be different for the biceps and triceps brachii. While corticospinal and spinal excitability to the biceps brachii were both higher during elbow flexion compared to extension, as expected, corticospinal excitability to the triceps brachii was not phase-dependent and spinal excitability was actually higher during elbow flexion than extension. These findings suggest that the neural control of these antagonistic muscle groups may be differentially controlled by supraspinal and spinal centres. These findings warrant further investigation to determine their underlying mechanisms.
